# A J-shaped relationship between caloric intake and survival in critically ill patients

**DOI:** 10.1186/s13613-015-0079-3

**Published:** 2015-11-05

**Authors:** Isabel Carolina Reis Crosara, Christian Mélot, Jean-Charles Preiser

**Affiliations:** Department of Intensive Care, Erasme University Hospital, Université Libre de Bruxelles, 808 route de Lennik, 1070 Brussels, Belgium; Department of Emergency Medicine, Erasme University Hospital, Université Libre de Bruxelles, Brussels, Belgium

**Keywords:** Nutrition, Nutritional support, Caloric intake, Glucose control, Insulin, Outcome

## Abstract

**Background:**

There is much controversy around the optimal caloric intake in intensive care unit (ICU) patients, based on the diverging results of prospective studies. Therefore, we assessed the presence of an association between caloric intake and outcome in a large cohort included in the Glucontrol study.

**Methods:**

Patients (n = 1004) were divided into four quartiles (q1–q4) according to the daily caloric intake (n = 251/quartile). ICU, hospital and 28-day mortality and the length of stay (LOS) in ICU and in the hospital were compared between each quartile, before and after adjustment in case of differences in baseline characteristics.

**Results:**

Caloric intake averaged 0.5 ± 0.6 (q1), 3.0 ± 0.7 (q2), 13.4 ± 5.1 (q3) and 32.4 ± 8.5 (q4) kcal/kg/day (*p* < 0.001 between quartiles). Comparisons among quartiles revealed that ICU, hospital and 28-day mortality were lower in q2 than in the other quartiles. ICU and hospital LOS were lower in q1 and q2. After adjustment for age, type of admission and severity scores, hospital mortality was lower in q2 than in the other quartiles, and LOS was lower in q1and q2 than in q3–q4.

**Conclusions:**

In this large and heterogeneous cohort of ICU short stayers, a J-shaped relationship between the amount of calories provided and outcome was found. These hypothesis generating findings are consistent with the concept of improved clinical outcome by early energy restriction.

Trial registration#: ClinicalTrials.gov# NCT00107601, EUDRA-CT Number: 200400391440

## Background

The optimal caloric intake in critically ill patients is a matter of intense debate [1–6]. Indeed, several retrospective studies reported a positive correlation between the magnitude of the caloric debt and the rate of complications [7–9], while others reported that the provision of 25–66 % of the recommended caloric intake was associated with the best outcome [10, 11]. Similarly, some interventional trials reported a worse outcome for the patients receiving the larger dose of calories (i.e. 25–30) than the smaller dose (10–15 kcal/kg/day) during the first days of the stay in the intensive care unit (ICU) [12, 13], while others were unable to find a clinically relevant difference between patients randomised to a high-caloric versus low-caloric nutrition therapy [1, 14–17], even though statistically significant differences in secondary and tertiary outcome variables were found for one study [15]. The uncertainty regarding the optimal caloric intake is reflected by important divergences between guidelines, especially regarding the timing of adding supplemental parenteral nutrition when enteral intake is deemed insufficient during the first seven days of the ICU stay [18, 19].

In fact, the requirements for nutrients are probably variable and mostly unpredictable in critically ill patients, related to the nutritional and inflammatory status at admission, the timing from admission, the metabolic changes related to the critical illness itself or the treatments. The caloric needs may vary over time, as a function of the actual energy expenditure (EE), and the proportion of EE met by the mobilisation of endogenous substrates. In particular, the non-inhibitable endogenous production of glucose can match a substantial proportion of the EE during the first few days of critical illness [4, 20] even though the endogenous glucose production cannot be assessed at bedside. Recently, the inhibition of autophagy was added to the list of potential mechanism underlying the deleterious effects of an excessive caloric intake [3].

With the question of the optimal caloric intake in mind, we aimed to address the following issue: Is there a relationship between the total amount of calories provided to critically patients and outcome, i.e. mortality and rate of complications reflected by the length of stays (LOS) in the ICU and in the hospital? To answer this question, we use the database of Glucontrol, a multi-centre trial of glycemic control which included a large heterogeneous population of medico-surgical ICUs of seven countries [21].

## Methods

The database of the Glucontrol study (ClinicalTrials.gov# NCT00107601, EUDRA-CT Number: 200400391440) was used for this post hoc analysis of data. The details of the design and conduct of this study have been published elsewhere [21]. In brief, this prospective study included 1078 patients admitted in 21 medical–surgical ICUs of 19 hospitals in Europe and Israel. Patients were randomised to a liberal glucose control strategy (target blood glucose 140–180 mg/dl) or to a tight glucose control strategy (target blood glucose 80–110 mg/dl). No difference in outcome was found between the two treatment arms, allowing the analysis of the whole set of data regardless of the randomisation. For the purpose of the present study, we calculated the caloric intake from the amount of calories provided by enteral nutrition, parenteral nutrition and non-nutritional solutions (i.e. total glucose amount administered intravenously). The amounts of proteins and calories provided by meals and by lipids used as solvent for sedative agents were not available. Patients for whom these data were missing were not included in this study. The daily caloric intake was calculated as the sum of calories expressed in kcal/kg (weight recorded at admission) divided by the number of days spent in the ICU. The patients were divided into four quartiles of increasing caloric intake (q1, q2, q3, q4), regardless of the treatment group. The caloric intake was also calculated for the first 5 days, for each quartile. Baseline characteristics including demographic data (age, gender), type of admission (medical, scheduled surgical, emergency surgical, non-surgical trauma), severity scores (Acute Physiological and Chronic Health Evaluation (APACHE) II [22] and Sequential Organ failure assessment (SOFA) [23]), body mass index (BMI) and treatment arm of the Glucontrol study were compared between the four quartiles of caloric intake. The percentages of patients with different categories of BMI (<20, 20–25, 25–30, 30–35, 35–40 and ≥40) were calculated for each quartile. In case of significant differences between groups for the baseline characteristics, an adjustment for these characteristics was planned. The outcome variables included ICU mortality, in-hospital mortality, 28-day mortality, LOS in the ICU and in the hospital, as indices of the rate of complications.

### Statistical analysis

The continuous variables are presented as mean ± SD or as median (interquartile range, IQR), based on the normality or non-normality of the distribution (Shapiro–Wilk test). The categorical variables are presented as percentages. For the comparison of the four quartiles, an analysis of variance parametric or non-parametric (Kruskal–Wallis test) was used followed by a pairwise comparison using either a modified *t* test or a Mann–Whitney–Wilcoxon test with a Bonferroni’s correction. A χ^2^ test was used to compare the percentage in different quartiles. When the quartiles were evaluated separately in both groups, an additional factor was added to ANOVA to take into account the different groups. An analysis of covariance (ANCOVA) was used to evaluate the different quartiles with adjustment for the divergent baseline characteristics, using these variables as covariates. A *p* value <0.05 was considered as significant. All statistical analyses were performed using Statistix 9.0.

## Results

A complete set of data was available for 1004 patients (93.1 % of the entire cohort). As planned, the cohort was divided into quartiles (q1–q4) according to the caloric intake during the ICU stay, irrespective of the treatment group.

### Baseline characteristics

The baseline characteristics of the patients of the four quartiles of caloric intake are shown in Table 1. These characteristics were similar between quartiles, except for age (higher in q2), type of admission (scheduled surgery more frequent in q2, emergency surgery and trauma more frequent in q4), severity scores (APACHE II and SOFA scores lower in q2, higher in q4). Because of these differences between quartiles, an adjusted analysis for age, type of admission, APACHE II and SOFA scores was carried out, in addition to the unadjusted analysis.

**Table 1:** Baseline characteristics

Admission data	q1 (*N* = 251)	q2 (*N* = 251)	q3 (*N* = 251)	q4 (*N* = 251)	*p* value
Age (years)	58 ± 18	67 ± 13	60 ± 17	58 + 18	<0.0001
Gender (% of males)	60 %	62.3 %	61.6 %	67.33 %	0.43
Type of admission (%)					
Medical	43.9 %	25.2 %	53.2 %	43.0 %	<0.0001
Scheduled surgery	32.9 %	64.0 %	21.0 %	15.1 %	<0.0001
Emergency surgery	12.6 %	10.4 %	17.7 %	29.9 %	<0.0001
Trauma	12.6 %	0.0 %	8.1 %	12.0 %	<0.0001
Admission APACHE II score (mean ± SD)	16.2 ± 6.9	13.2 ± 5.8	17.4 ± 6.8	19.2 ± 7.3	<0.0001
SOFA score (mean ± SD)	5.9 ± 4.0	4.8 ± 2.0	6.0 ± 2.8	6.9 ± 2.3	<0.0001
BMI (kg/m^2^) (%)					0.11
<20	5.4 ± 1.4	2.5 ± 1.4	4.0 ± 1.4	6.9 ± 1.4	
20–25	28.4 ± 3.2	34.0 ± 3.0	28.2 ± 3.1	42.0 ± 3.1	
25–30	38.7 ± 3.3	37.7 ± 3.1	37.9 ± 3.2	35.9 ± 3.2	
30–35	20.7 ± 2.6	20.9 ± 2.5	20.7 ± 2.6	11.3 ± 2.5	
35–40	3.2 ± 1.3	4.1 ± 1.2	4.9 ± 1.3	3.0 ± 1.3	
>40	3.6 ± 1.0	0.8 ± 1.0	4.4 ± 1.0	0.9 ± 1.0	
Weight (kg)	77 ± 17	77 ± 15	80 ± 18	72 ± 13	
% of patients from glucontrol study					0.92
Liberal glucose control group	50.6	47.6	49.4	49.8	
Tight glucose control group	49.4	52.3	51.6	51.2	

*BMI* body mass index

### Nutritional variables

The caloric requirements were slightly but significantly lower in q4 than in the other quartiles (Table 2). As expected, the amount of calories provided steadily increased from q1 to q4, even when limited to the first 5 days (Tables 3, 4). Of note, the majority of calories provided in q1 and q2 were given as intravenous non-nutritional glucose solutions. The average blood glucose concentrations were similar in each quartile.

**Table 2:** Nutritional data

	q1 (*N* = 251)	q2 (*N* = 251)	q3 (*N* = 251)	q4 (*N* = 251)	*p* value
Daily caloric expenditure (25 kcal/kg/day)	1923 ± 437	1915 ± 370	1995 ± 443	1800 ± 337	<0.0001
Average total caloric intake (kcal/kg/day)	39 ± 53	226 ± 58	1082 ± 462	2325 ± 702	<0.0001
Total percentage of energy intake/requirement (%)	1.9 ± 2.6	12.0 ± 3.0	53.8 ± 20.6	129.7 ± 34.0	<0.0001
Calories given enterally (kcal/kg/day)	0	0.02 ± 0.25	8.30 ± 7.04	18.21 ± 9.19	<0.0001
Calories given parenterally (kcal/kg/day)	0	0	2.82 ± 5.41	11.73 ± 10.16	<0.0001
IV glucose in non-nutrition solutions (g)	9.72 ± 13.31	55.91 ± 14.49	43.15 ± 33.00	43.25 ± 29.62	<0.0001
Average blood glucose concentrations (mg/dL)	133 ± 30	133 ± 24	135 ± 28	134 ± 28	0.8618

*IV* intravenous

**Table 3:** Nutritional data (first 5 days)

	q1 (*N* = 251)	q2 (*N* = 251)	q3 (*N* = 251)	q4 (*N* = 251)	*p* value
Daily caloric expenditure (25 kcal/kg/day)	1923 ± 437	1915 ± 370	1995 ± 443	1800 ± 337	<0.0001
Average total caloric intake (kcal/kg/day)	185 ± 456	230 ± 62	940 ± 471	1653 ± 615	<0.0001
Total percentage of energy intake/requirement (%)	9.9 ± 2.5	12.2 ± 3.2	47.3 ± 22.9	93.4 ± 35.1	<0.0001
Calories given enterally (kcal/kg/day)	0.62 ± 2.93	0.04 ± 0.36	6.73 ± 6.67	11.19 ± 9.66	<0.0001
Calories given parenterally (kcal/kg/day)	1.27 ± 4.24	0	2.74 ± 5.64	9.83 ± 10.38	<0.0001
IV glucose in non-nutrition solutions (g)	11.60 ± 22.09	56.72 ± 14.81	43.70 ± 34.28	40.64 ± 41.52	<0.0001
Average blood glucose concentrations (mg/dL)	134 ± 32	134 ± 24	138 ± 32	138 ± 30	0.1773

**Table 4:** Outcome data

	q1 (*N* = 251)	q2 (*N* = 251)	q3 (*N* = 251)	q4 (*N* = 251)	*p* value
Mortality (%)					
ICU	15.14	5.18	16.73	22.31	<0.0001
Hospital	21.51	6.37	24.30	28.29	<0.0001
LOS (days)					
ICU	4 (2–6)	4 (3–5)	8 (4–13)	15 (9–23)	<0.0001
Hospital	13 (8– 3)	12 (10–16)	19 (11–31)	28 (17–41)	<0.0001
Infection during ICU stay (%)					
Antibiotics	9.16	18.33	65.74	84.46	<0.0001
Positive blood culture	1.59	6.77	25.10	20.32	<0.0001

Median (interquartile range)

*LOS* length of stay, *ICU* intensive care unit

### Outcome variables

#### Mortality

**Fig. 1:**
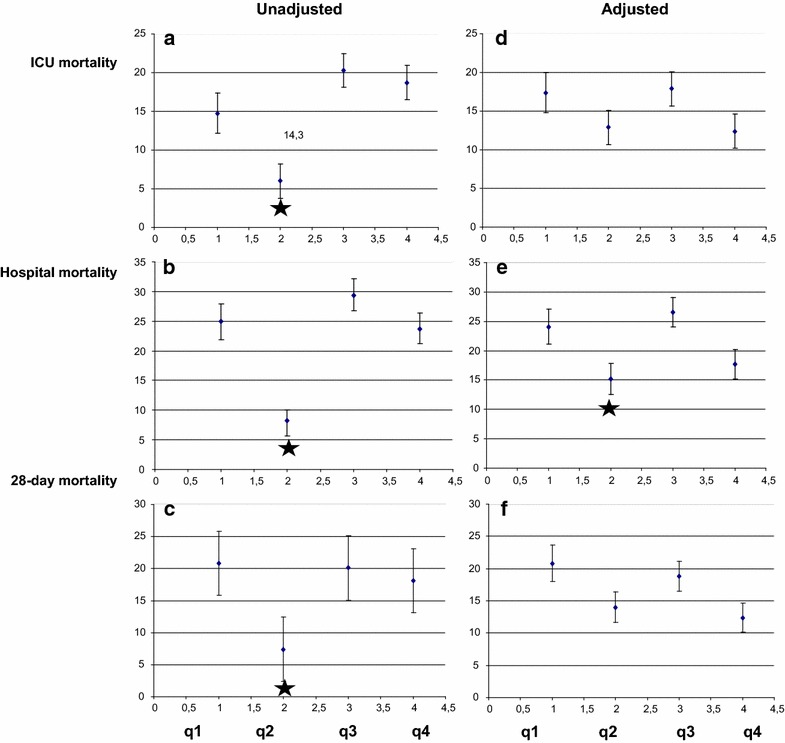
ICU, hospital and 28-day mortality of the four quartiles of caloric intake, expressed in percentages before (**a** *–* **c**) and after (**d** *–* **f**) adjustments for between-quartile differences in baseline characteristics (age, type of admission, APACHE II and SOFA scores). The *star* denotes a statistically significant difference (*p* < 0.05)

The global ICU, in-hospital and 28-day mortality rates were 14.8 ± 1.1, 21.4 ± 1.3 and 16.4 ± 1.2 %, respectively. Comparison between quartiles revealed that the unadjusted rates of mortality in the ICU, in the hospital and at 28 days were lower in patients of q2 than in the other quartiles (Fig. 1a–c), yielding a J-shaped curve. After adjustment for the type of admission, age, APACHE II and SOFA scores, the hospital mortality rate was still lower in the q2 than in the others (*p* < 0.05, Fig. 1e), while ICU and 28-day mortality were non-significantly lower in q2 than in the other quartiles.

#### Length of stays

**Fig. 2:**
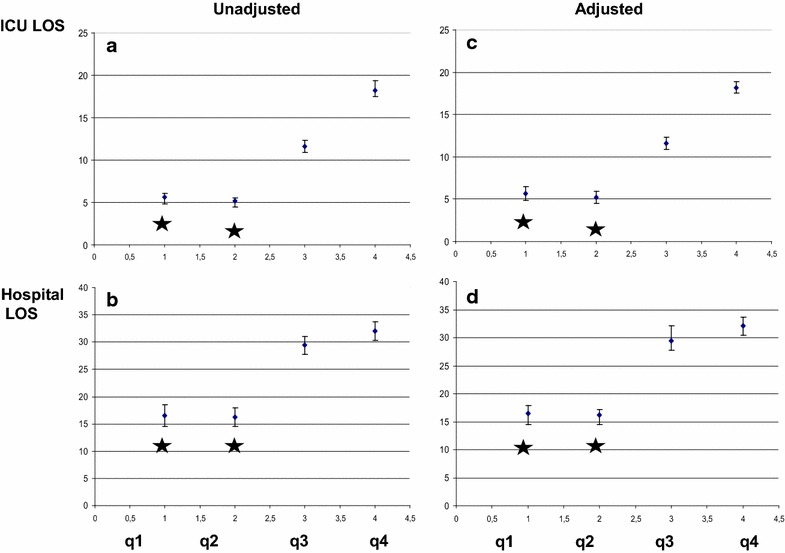
ICU and hospital length of stay (days) of the four quartiles of caloric intake, before (**a**, **b**) and after (**c**, **d**) adjustments for between-quartile differences in baseline characteristics (age, type of admission, APACHE II and SOFA scores). The *star* denotes a statistically significant difference (*p* < 0.05)

The global LOS in the ICU and in the hospital of the cohort of patients averaged 6 [3–13] and 16 [11–29] days, respectively. Comparison between quartiles revealed that the unadjusted LOS in the ICU and in the hospital were lower in patients of q1 and q2 than in the q3 and q4 (Fig. 2a, b, *p* < 0.0001). After adjustment, both LOS in ICU and in the hospital were still lower in the q1 and q2 than in q3 and q4 (*p* < 0.0001, Fig. 1c, d).

## Discussion

In this post hoc analysis of data of a large cohort of critically ill patients, the highest survival rate was surprisingly found in patients who received 25–50 % of their presumed caloric requirements (q2), as compared to those receiving higher and lower caloric intake. Consistently, the LOS, considered as a surrogate marker of the rate of complications, was also found lower in patients who received the lower amount of calories, possibly related to a higher rate of infections in q3 and q4. Alternatively, the shorter LOS found in q2 could underlie the lower caloric intake, when patients getting better, for instance after uncomplicated surgery are more likely to eat and to be discharged earlier. Nevertheless, these associations even held true after adjustment for the differences in baseline characteristics.

The strengths of this study include the large size of the sample, the wide variety of admission diagnoses, the adjustment for baseline differences and the short duration of stay in the ICU, which is typical of medico-surgical ICUs worldwide. The external validity of these findings can be further supported by the patients’ characteristics (age around 60 years, predominance of males, severity scores, mortality rate). Remarkably, most of the calories in q1 and q2 were provided via non-nutritional solutions. Admittedly, the comparability between quartiles was limited by the shorter LOS of patients of q1 and q2 than q3 and q4, thereby reducing the likelihood to receive any form of artificial nutrition. Similarly, some severity factors, such as nutrition status or ability of patients to eat could have influenced the prescription of nutrition and the outcome. Importantly, BMI did not differ between groups, preventing any extrapolation over the effects of caloric intake as a function of the prior nutritional status, in contrast to a former retrospective study [7].

In a similar set of short stayers, Casaer [13] and Doig [15] found no advantage or even deleterious effects of an additional provision of calories by supplemental parenteral nutrition when enteral nutrition was deemed insufficient [2, 4]. Likewise, in medical patients, Krishnan [10] and Rubinson [11] found no benefit of a caloric intake calculated to match the recommended target of 27.5 kcal/kg/day. The results of the present study are also consistent with those of retrospective large-scale and prospective studies, in which the provision of large amounts of calories was associated with a poorer outcome than the provision of smaller amounts [10, 11], and in contrast with reports that indicated a relationship between the magnitude of caloric debt and the rate of complications in long stayers [7, 8], i.e. patients who stayed in the ICU longer than one week. Importantly, there was no difference in the average blood glucose levels between quartiles, probably owing to the similar distribution of patients randomised into the two experimental groups of Glucontrol within each quartile (Table 1).

There are also limitations of this study, including the lack of data for 6.9 % of the study participants, its post hoc design, related to the lower interest brought by the community of intensivists to the issue of optimal caloric intake at the time of designing Glucontrol [21]. The absence of details related to the daily caloric provision, the protein and dietary intakes represent clear weaknesses, especially when few calories were administered enterally. However, the oral intake of critically ill patients is often limited [24] and infusion of short-lived lipophilic sedative agents (i.e. propofol) is typically short.

Several explanations can be suggested for these findings, including the presence of occult overfeeding when exogenous calories are delivered on top of the non-inhibitable endogenous source. Indeed, the non-inhibitable production of glucose by gluconeogenic organs provides a substantial amount of calories during the acute phase of critical illness [20]. The anorexia of acute illness might reflect this physiological mechanism [25], via the gut–brain axis [26]. The inhibition of autophagy is another possible explanation [4, 27, 28]. From a clinical standpoint, the results of this study further support updated recommendation to limit the caloric deficit rather than trying to compensate it, during the early phase [29]. Future studies should probably aim to quantify the non-inhibitable endogenous caloric production, to estimate the actual caloric needs. The beneficial effect of matching these needs on the outcome variables would then be confirmed by prospective interventional trials.

In conclusion, the importance of the amount of daily calories provided during critical illness has been previously under estimated. The caloric requirements could be lower during the early than during the late phase of critical illness, implying that the determination of the caloric needs by the assessment of EE is meaningful in long stayers [4]. In contrast, matching the caloric needs based on EE can lead to overfeeding during the acute early phase.
